# Metastatic Renal Cell Carcinoma Presenting as a Paranasal Sinus Mass: The Importance of Differential Diagnosis

**DOI:** 10.1155/2017/9242374

**Published:** 2017-01-11

**Authors:** Massimo Ralli, Giancarlo Altissimi, Rosaria Turchetta, Mario Rigante

**Affiliations:** ^1^Department of Oral and Maxillofacial Sciences, Sapienza University of Rome, Rome, Italy; ^2^Department of Sense Organs, Audiology Section, Policlinico Umberto I, Sapienza University of Rome, Rome, Italy; ^3^Department of Otorhinolaryngology, Catholic University of Sacred Heart, Rome, Italy

## Abstract

Metastases in the paranasal sinuses are rare; renal cell carcinoma is the most common cancer that metastasizes to this region. We present the case of a patient with a 4-month history of a rapidly growing mass of the nasal pyramid following a nasal trauma, associated with spontaneous epistaxis and multiple episodes of hematuria. Cranial CT scan and MRI showed an ethmoid mass extending to the choanal region, the right orbit, and the right frontal sinus with an initial intracranial extension. Patient underwent surgery with a trans-sinusal frontal approach using a bicoronal incision combined with an anterior midfacial degloving; histological exam was compatible with a metastasis of clear cell renal cell carcinoma. Following histological findings, a total body CT scan showed a solitary 6 cm mass in the upper posterior pole of the left kidney identified as the primary tumor. Although rare, metastatic renal cell carcinoma should always be suspected in patients with nasal or paranasal masses, especially if associated with symptoms suggestive of a systemic involvement such as hematuria. A correct early-stage diagnosis of metastatic RCC can considerably improve survival rate in these patients; preoperative differential diagnosis with contrast-enhanced imaging is fundamental for the correct treatment and follow-up strategy.

## 1. Introduction

Renal cell carcinoma (RCC) is the most common kidney cancer, with approximately 35,000 new cases in the US each year [1]; RCC mainly affects male patients between 40 and 60 years old [2]. Common presentation symptoms include hematuria (40%), flank pain (40%), and a palpable abdominal mass (25%) [3]. Approximately 30% of patients with renal cell carcinoma present with metastatic disease [4]; target organs are lung (75%), soft tissues (36%), bone (20%), liver (18%), cutaneous sites (8%), and central nervous system (8%) [5, 6]. Metastases in the paranasal sinuses are rare [7]; however, RCC is the most common cancer that metastasizes to this region. Prognosis of metastatic RCC is poor [8]; the survival rate ranges between 15 and 30% at 5 years [9] in case of a single metastasis and between 0 and 7% in patients with multiple metastases [10]. Metastatic RCC is often resistant to chemotherapy and radiotherapy [11]; numerous agents targeting VEGF and non-VEGFR pathways have been proposed during the last decade for the treatment of advanced RCC [12–18].

We present the case of a patient with a single, rapidly growing mass in the upper portion of the nasal pyramid, with late, postnasal surgery histological diagnosis of renal cell carcinoma that allowed primary tumor identification.

## 2. Case Presentation

**Figure 1:**
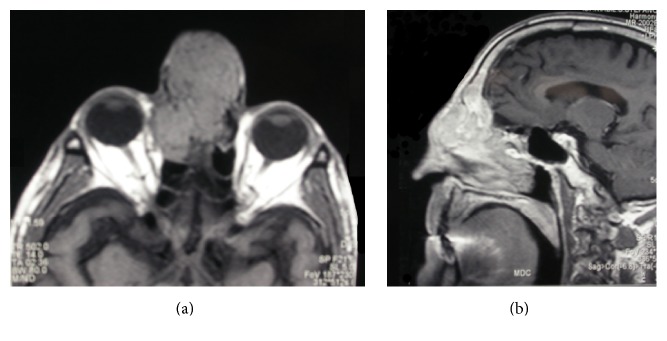
MRI in the axial (a) and sagittal (b) planes showing a soft tissue ethmoid mass extending to the right and left choanal region, the right orbit, the right frontal sinus, and an initial intracranial extension with partial erosion of the crista galli.

A 72-year-old man was referred to our institution with a 4-month history of a voluminous mass in the upper portion of the nasal pyramid following a nasal trauma. He had been treated a few weeks earlier at a different ENT service for a massive spontaneous epistaxis. The patient also reported a long history of hematuria, previously attributed to renal tuberculosis occurring over 40 years before. At admission, a cranial CT scan showed a large soft tissue ethmoid mass extending to the right and left choanal region, the right orbit, the right frontal sinus, and an initial intracranial extension with partial erosion of the crista galli. MRI confirmed the evidence found at computed tomography (Figure 1). Fine needle aspiration showed typical epithelial tissue and clear-cytoplasm cells interpreted as pericytes. Preoperative local biopsy was not performed due to the history of severe epistaxis and the high risk of massive bleeding during the procedure.

**Figure 2:**
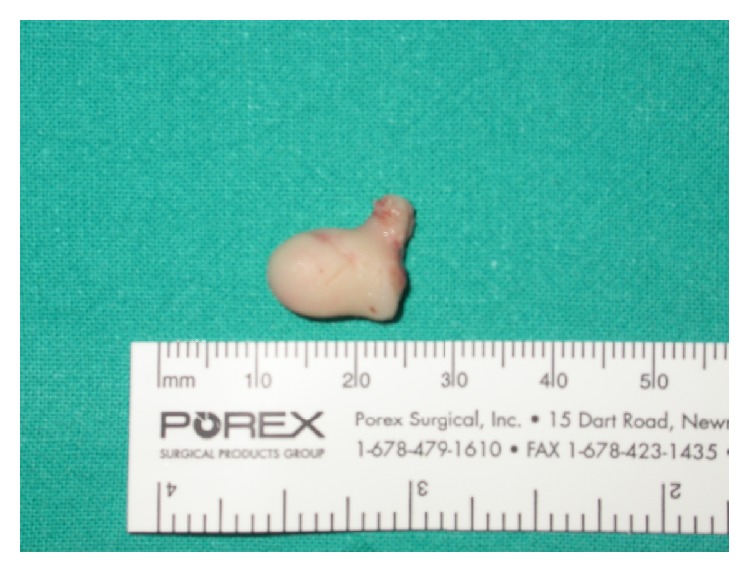
The excised mass; histological exam was consistent with a clear cell renal cell carcinoma.

The patient underwent surgery with a trans-sinusal frontal approach using a bicoronal incision combined with an anterior midfacial degloving to excise the mass; however, the right orbital and especially the initial intracranial extension did not allow a complete removal of the neoplasm. Considerable bleeding occurred during surgery. The histological exam revealed a clear cell renal cell carcinoma (Figure 2). Based on these findings, the patient underwent a total body CT scan that showed a solitary 6 cm mass in the upper posterior pole of the left kidney. Bone scintigraphy also revealed increased uptake in the ethmoid and orbital region. Due to the poor general conditions, no surgery was performed to remove the primary tumor; the patient died 4 months later.

## 3. Discussion

Nasal cavity and paranasal sinus cancers are usually primary tumors. Metastases to the paranasal sinuses are rarely found; among them, renal cell carcinoma is the most common cancer to metastasize to this region (49%) followed, respectively, by bronchus, urogenital ridge, breast, and gastrointestinal tract [19, 20]. RCC can metastasize to any region of the body, with a prevalence for lungs (75% of cases), regional lymph nodes (65%), bone (40%), and liver (40%) [21]. Metastasis to the head and neck regions account for about 15% of the cases, targeting in order of frequency the paranasal sinuses, the larynx, jaws, temporal bones, thyroid, and parotid glands [22].

RCC tumor cells can reach the sinonasal region via two routes: the first includes inferior vena cava, lungs, heart, and the maxillary artery; the second involves the communication of the avalvular vertebral venous plexus and the intracranial venous plexus [23]. Maxillary sinuses are the most commonly involved sinuses by metastatic tumors (36%), followed by the ethmoid (25%), frontal and sphenoid sinuses (17%), and nasal cavity (11%) [24, 25]. One of the first reports available in recent literature to describe a renal clear cell carcinoma metastatic to the paranasal sinuses has been published by Matsumoto and Yanagihara in 1982 [26]; afterwards several authors described case reports of RCC presenting as metastatic diseases in the paranasal sinuses. Available literature describes presentation of RCC metastasis as a solitary periorbital [27] and orbital mass [28], as a frontal sinus mass [29], as an ethmoid sinus mass [30, 31], in the nasal cavity [32, 33], in the maxillary [34, 35], and sphenoid sinus [36–38]. In some cases, the extension of the metastasis to the skull base has been described [39].

Metastatic RCC to the sinonasal district has been reported as the presenting sign of this disease in a few cases [29, 34], while in others it followed or occurred simultaneously to primary cancer diagnosis. Presentation symptoms are often limited to recurrent epistaxis [40–43] and the presence of a primary renal cell carcinoma is recognized only after surgical removal of the metastatic tumor via histologic examination supported by immunohistochemical staining of the specimen [5]. Rarely, metastasis in the sinonasal cavities followed RCC diagnosis and treatment [44–46]; cases of postsurgery metastasis in the head and neck district have been described up to 12 years after surgery [47].

The key point in RCC presenting with a sinonasal metastasis is differential diagnosis with primary tumors such as adenocarcinomas, angiofibromas, hemangiopericytomas, melanomas, hemangiomas, metastatic tumors from the breast and lungs, and, more rarely, systemic diseases such as Wegener's and midline granulomas [48]. In fact, in such cases diagnostic delays, misdiagnosis, undertreatment, and mismanagement could occur due to (1) the attribution of the mass to a primary sinonasal cancer given the rare nature of sinonasal metastasis or (2) to the overlook of presenting symptoms such as recurrent epistaxis, swelling, pain, and nasal obstruction. Hematuria can be considered as an indicator of RCC; it has been reported that about 10% of patients with RCC with distant metastasis exhibit massive hematuria. However, intermittent hematuria may be present in 90% of cases [3]. For this reason, patients presenting with nasosinusal tumors also reporting hematuria should always undergo systemic evaluation. Radiological examination with CT scan and, secondly, MRI and angiography are necessary in assessing the extent of the metastatic lesion. However, it should be considered that RCC metastases have similar radiological appearances to primary malignant lesions of sinonasal cavities; some indicators of renal origin at CT scan are enhancement, destruction, and lack of tumoral calcification [6].

In this case, CT scan allowed the identification of a neoformed paranasal sinus mass; however, only histological exam identified the mass as a metastasis of RCC and led to the execution of total body CT scan to identify primary tumor. Although difficult, differential preoperative diagnosis is fundamental for the correct treatment and follow-up strategy; contrast-enhanced imaging plays a central role since a preoperative biopsy of the nasal mass may be difficult in these patients due to massive recurring bleeding and, in some cases, may result in only necrotic tissue inconclusive on histopathology [42]. The ENT specialist, therefore, should always suspect metastatic disease from primary sites external to the head and neck region in patients with hypervascular mass in the nasal cavity or paranasal sinuses and a history of massive nasal bleeding and should complete preoperative workup with total body CT scan. Furthermore, it is important to remark that metastatic tumors originating from primary kidney masses are highly vascularized and surgeons should expect significant haemorrhage during surgical removal. One of the main advantages of a preoperative diagnosis of RCC when approaching a patient with sinonasal mass is the preparation for management of severe perioperative bleeding, thus implementing strategies to optimise the patient's tolerance to bleeding and to reduce the amount of bleeding morbidity and mortality.

Prognosis of metastatic RCC is poor; however, a correct early-stage diagnosis of metastatic disease can considerably improve survival rate: literature reports that excision of solitary metastatic lesion of renal cell carcinoma following nephrectomy results in a 41% survival at 2 years and 13% survival at 5 years [48]. The sole excision of the metastatic lesion, instead, significantly lowers survival rate [49]; patients with multiple metastases have a 5-year survival rate between 0 and 7% [10].

Although metastatic RCC is often resistant to chemotherapy and radiotherapy, numerous agents targeting VEGF and non-VEGFR pathways should be taken into account for the treatment of advanced RCC. Multitargeted VEGF tyrosine kinase inhibitors (TKIs) include sorafenib [12], sunitinib [13], pazopanib [14], axitinib [15], and bevacizumab [16]; mTOR inhibitors include temsirolimus [17] and everolimus [18]. Unfortunately, especially in cases of advanced neoplasms, benefits are still time-limited and treatment decisions should be based not only on guidelines but also on clinical considerations, such as patient comorbidities, treatment toxicity, prognostic factors, and molecular aspects of disease. In this case, the poor general conditions of the patient prevented additional treatment except for palliative pain management.

In conclusion, metastatic renal cell carcinoma should always be suspected in patients with nasal or paranasal masses, especially if associated with symptoms suggestive of a systemic involvement such as hematuria; early-stage diagnosis of metastatic disease can considerably limit perioperative complications and improve survival rate.
